# Parallel damage in mitochondrial and lysosomal compartments promotes efficient cell death with autophagy: The case of the pentacyclic triterpenoids

**DOI:** 10.1038/srep12425

**Published:** 2015-07-27

**Authors:** Waleska K. Martins, Érico T. Costa, Mário C. Cruz, Beatriz S. Stolf, Ronei Miotto, Rodrigo M. Cordeiro, Maurício S. Baptista

**Affiliations:** 1 Instituto de Química, Universidade de São Paulo, Brazil; 2 Ludwig Institute for Cancer Research (LICR) at Centro de Oncologia Molecular, Hospital Sírio Libanês, São Paulo, Brazil; 3 Instituto de Ciências Biomédicas, Universidade de São Paulo, Brazil; 4 Centro de Ciências Naturais e Humanas, UF-ABC, Brazil

## Abstract

The role of autophagy in cell death is still controversial and a lot of debate has concerned the transition from its pro-survival to its pro-death roles. The similar structure of the triterpenoids Betulinic (BA) and Oleanolic (OA) acids allowed us to prove that this transition involves parallel damage in mitochondria and lysosome. After treating immortalized human skin keratinocytes (HaCaT) with either BA or OA, we evaluated cell viability, proliferation and mechanism of cell death, function and morphology of mitochondria and lysosomes, and the status of the autophagy flux. We also quantified the interactions of BA and OA with membrane mimics, both *in-vitro* and in-silico. Essentially, OA caused mitochondrial damage that relied on autophagy to rescue cellular homeostasis, which failed upon lysosomal inhibition by Chloroquine or Bafilomycin-A1. BA caused parallel damage on mitochondria and lysosome, turning autophagy into a destructive process. The higher cytotoxicity of BA correlated with its stronger efficiency in damaging membrane mimics. Based on these findings, we underlined the concept that autophagy will turn into a destructive outcome when there is parallel damage in mitochondrial and lysosomal membranes. We trust that this concept will help the development of new drugs against aggressive cancers.

Macroautophagy, or simply autophagy, is a lysosome-dependent degradation pathway that promotes cell homeostasis in response to several types of stresses^1^. After decades of scientific discoveries^2^ the general agreement is that the protective role of autophagy may be converted into a destructive one, i.e., autophagy associates with cell death when there is failure in either the fusion of autophagosomes with lysosomes or in the digestion of autolysosomes^3^. However, the understanding of this process at the molecular level needs a profound analysis of the competition between the activation and inhibition pathways of autophagy. Consequently, the impact of activating autophagy with damaged mitochondria (mitophagy) on the condition of autophagy impairment by lysosome damage is a noteworthy subject to explore. If mitophagy fails, the decrease in removal of injured mitochondria lead to accumulation of enlarged mitochondria, cell aging, genomic instability and senescence^4^,^5^,^6^,^7^.

In here, we report a comparative study of the biological effects of two chemical isomers, the pentacyclic triterpenoids Betulinic (BA) and Oleanolic (OA) acids, in a cellular model of immortalized human skin keratinocytes (HaCaT)^8^, in which homeostasis strictly depends on autophagy pathway^9^. Consequently, in HaCaT it is possible to detect endogenous LC3 lipidated form (LC3-II)^10^, avoiding artifacts that may occur when employing transfection and transgenesis strategies^11^.

As expected, BA and OA are almost identical in terms of their physicochemical properties (Table 1), however they significantly differ in cytotoxicity, an effect that has not yet been properly explained^12^,^13^. BA is highly toxic to cells^12^,^13^,^14^, which the literature attributes mainly to activation of apoptosis by mitochondrial damage^15^,^16^,^17^,^18^,^19^.

Autophagy has been activated upon BA treatment in an attempt to retard mitochondria-mediated apoptosis in tumors cells^18^. Once suppressed, autophagy fails to guarantee cell recovery, and a significant increase in apoptosis BA-modulated was shown to take place in human multiple myeloma cells^20^. However, it is still unknown how BA interplays the mitochondrial-lysosomal axis of autophagic cellular rescue. Gonzalez *et al.* also reported that BA derivative B10 is capable of inducing cell death mainly by inhibition of the autophagic flux through the release of cathepsins (B and Z) in the cytosol^21^. Although both studies^20^,^21^ observed undigested autophagosomes, the connection between lysosomal membrane impairment and autophagy effectiveness was not addressed.

OA is widely used as an anti-inflammatory, antiangiogenic and antioxidant agent^13^,^22^. At large concentrations, OA also induces mitochondrial damage leading to apoptotic cell death^23^. However, there are no reports of autophagy induction or cell death with autophagy upon OA treatment.

Motivated by the lack of mechanistic explanation and by contradictory data in the literature concerning the BA mechanism of cell death^18^,^20^,^21^, we investigated the role of autophagy on the underlying biological processes triggered by BA and OA. By comparing the responses of BA with OA, we were able to reveal details of the induction and inhibition of the autophagic process and their association with cell death and damage in mimetic membranes.

## Results

The main experimental procedure in this work consisted in incubating HaCaT with BA, OA and other chemicals. We reported the results referring to the time at which the experiment was performed, after the incubation with chemicals. The label T1 was used for experiments performed just after the 24-hour incubation period. T2 and T3 referred to experiments performed 24 and 48 hours after T1. Experiments performed at other temporal schemes will be clearly identified throughout the text.

### BA is more cytotoxic than OA in HaCaT

**Figure 1:**
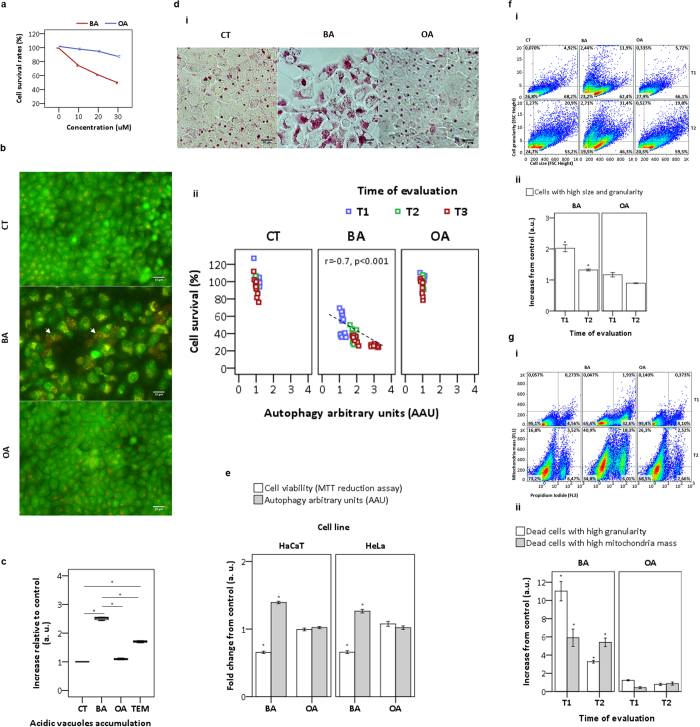
Analysis of biological effects of BA and OA. (**a**) Survival rates at T3 as a function of dose (10–30 μM). (**b**) At T1 with DMSO (control) and triterpenoids (20 μM), HaCaT keratinocytes were stained with the dyes Acridine Orange (AO) and Propidium Iodide (PI), and evaluated under microscope. Arrows indicated nuclear PI positive dead cells with acidic vacuoles accumulation. (**c**) Alternatively, at T2 with DMSO (control), triterpenoids (20 μM) or TEM (15 μM) the acidic vacuoles AO-stained were quantified by FACS. (**d**) At T2 HaCaT keratinocytes treated with DMSO (control) and triterpenoids (20 μM) following staining with Neutral Red (30 μg/mL) were evaluated under microscope (**i**). At indicative times, the cell survival was assayed by MTT and linearly correlated to lysosomal content measured in terms of autophagy arbitrary units – AAU (**ii**). (**e**) At T3 with DMSO (control) and triterpenoids (20 μM) the cell survival of HaCaT and HeLa treated cells was assayed by MTT reduction following AAU calculation. (**f**) At T1 and T2, HaCaT keratinocytes treated with DMSO (control) and triterpenoids (20 μM) following FACS were gated according the Side Scatter (SSC) and Forward Scatter (FSC) parameters (**i**). After gating, bars showed the ponderation of cell size (FSC) and granularity (SSC) compared to control and represented as arbitrary units (**ii**). (g) After the same experimental condition (**f**), a pseudo-color scatter-plots showed gating of HaCaT treated cells according to two parameters (mitochondrial mass and cell death), following staining with MitoTracker green FM and PI dye inclusion (**i**). Regarding these parameters, subpopulations of treated-cells were identified, weigthed by control and represented as arbitrary units (**ii**). All results were obtained from at least three independent experiments and expressed as mean values ± standard error. Multiple statistical comparisons were calculated by ANOVA test, and the p-value for each pairwise group was determined by Dunnett’s T3 (high variance between groups) or Bonferroni (low variance between groups) post-hoc test. The analysis of correlation was done using Pearson’s coefficient (r). Significance difference (p < 0.05) was depicted by asterisk.

BA, which was much more damaging to HaCaT than OA (Fig. 1a), had its cytotoxic effects associated with lysosomotropic vacuolization as revealed in live microscopy (Fig. 1b). Only after BA (and not after OA) we observed a conspicuous vacuolization in HaCaT at T1, which seems to end up in cell death - note Propidium Iodide (PI) incorporation in some BA-treated cells. Interestingly, the vacuolization observed in BA-treated cells (Fig. 1b) perceived even at T2, as revealed in flow cytofluorometric analysis (Fig. 1c). BA treated cells showed an accumulation of acidic vacuoles (Fig. 1c) 2.5 times larger than in control and in OA treated cells; and 1.5 times larger than in cells treated with Temsirolimus (TEM)^10^, which inhibits the mammalian target of rapamycin (mTOR) and consequently activates autophagy. Live microscopy following staining with Neutral Red (NR) also revealed at T2 a remarkable lysosomotropic vacuolization in BA-treated cells (and not in cells treated with OA) (Fig. 1d-i).

In an attempt to further elucidate the impact of BA and OA treatment on cellular homeostasis, we analyzed the correlation between the lysosomotropic vacuolization and cell viability according to a recent strategy of quantifying autophagy by arbitrary units (AAU, based on weighting NR-uptake by the average of cell survival)^10^. Note that in BA-treated cells there is a clear correlation between the decrease in cell viability and the increase in AAU. This correlation was observed neither in control nor in OA-treated cells (Fig. 1d-ii). The same toxic effects of BA on HaCaT were also observed in HeLa cells. Note that BA caused a considerable increase in cell death (30%) with increase in lysosomal content and consequently in AAU (30%) (Fig. 1e). OA did not induce toxicity in HeLa cells.

We also evaluated cell vacuolization triggered by BA by using the FSC and SSC parameters, which allow the identification of subsets of cells based on size and internal complexity (Fig. 1f). Note the increase in the subset of HaCaT with cell enlargement and granularity (upper right quadrant) at both T1 and T2 (Fig. 1f-ii). Unlike control and OA, under BA these parameters showed an increase of 2.0 and 1.3-fold at T1 and T2, respectively (Fig. 1f-ii). These results indicated a clear propensity for BA-treated cells to die in parallel with the accumulation of acidic vacuoles.

Next, we investigated the status of mitochondria after triterpenoid treatment. By gating mitochondria mass (MTG) as a function of cell death (PI incorporation), we observed (upper left quadrant at T2) significant mitochondrial accumulation in a subset of BA surviving cells (40.9%) in comparison to control (16.8%) and OA (26.3%) (Fig. 1g-i). Unlike OA, BA dead cells also showed mitochondrial accumulation (mean of 5.6-fold) with remarkable increase in cell granularity of 11.0 and 3.3-fold at T1 and T2, respectively (Fig. 1g-ii). The accumulation of mitochondria at T2 in BA-treated cells (both alive and dead), suggests the possible involvement of mitochondria on this scenario of cell death.

### Depolarized mitochondria triterpenoids-triggered activates autophagy

**Figure 2:**
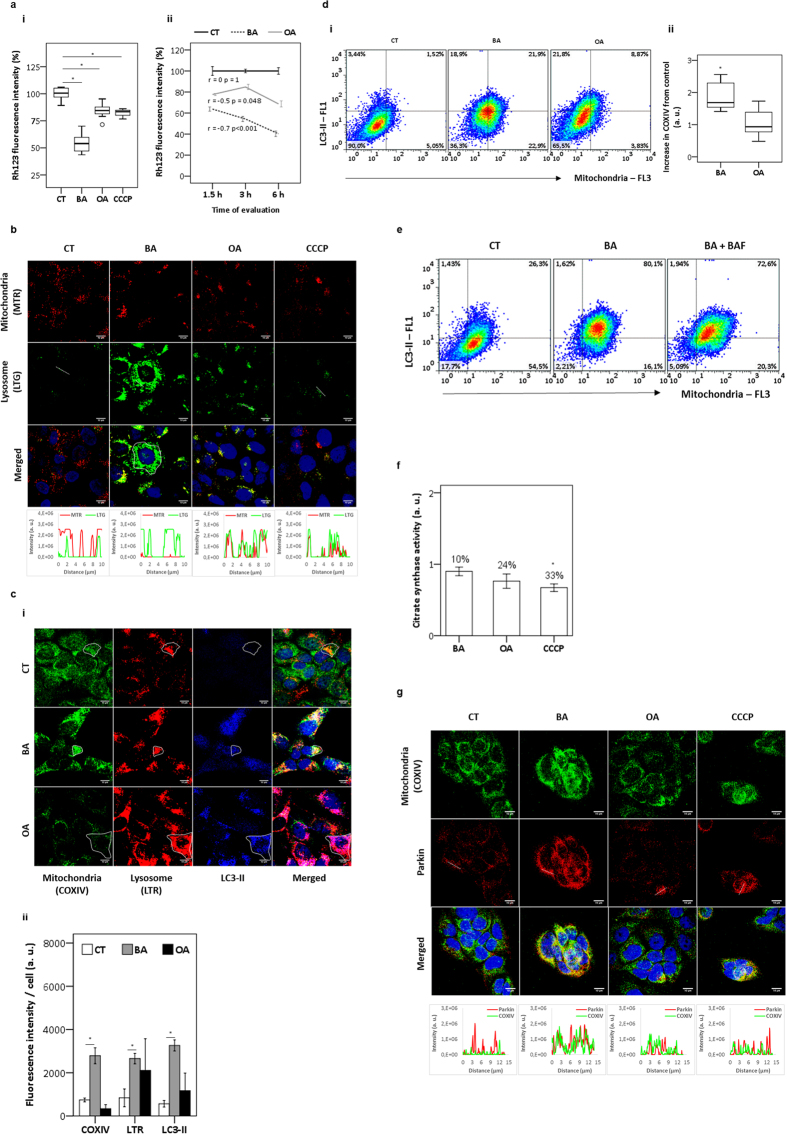
Analysis of mitochondrial membrane impairment in HaCaT. Keratinocytes were treated with DMSO (control), triterpenoids (20 μM), or CCCP (2 μM). (**a**) After 3 hours, the decrease in mitochondrial inner transmembrane potential (ΔΨm) was measured in terms of Rh123 fluorescence intensity relative to control (100%) (**i**). The decrease in Rh123 fluorescence intensity was evaluated as a function of time (**ii**). (**b**) Following staining for lysosomes with LTG (green) and mitochondria with MTR (red), cells were treated for 6 hours. Line scans (at bottom) indicated association between lysosome and mitochondria. This relationship represented the lines drawn in the images. (**c**) Keratinocytes were treated with DMSO (control), triterpenoids (20 μM). Following staining of lysosome with LTR (red), cells were immunostained for LC3-II (blue) and for the mitochondrial marker COXIV (green) at T1 (**i**). Bars showed the average of fluorescence intensity from multiple images (**ii**). (**d**) Scatter-plots of HaCaT following immunostaining for LC3-II (FL1) and COXIV (FL3) at T1 (**i**). Mitochondria mas (COXIV) compared to control and represented as arbitrary units (**ii**). (**e**) Scatter-plots at T1 showing LC3-II and COXIV in treated cells in presence or absence of BAF (20 ηM) added at the last two hours of the treatment. Keratinocytes treated with DMSO (control), triterpenoids (20 μM), or CCCP (2 μM) for 6 hours, following (**f**) citrate synthase (CS) activity represented as arbitrary units after ponderation to control; and (**g**) immunostaining for COXIV (green) and Parkin (red). At bottom, plot profile (Parkin/COXIV) of lines drawn in the images. T1 = after treatment for 24 hours; T2 = at 24 hours after T1; T3 = at 48 hours after T1. All results were obtained from at least three independent experiments and expressed as mean values ± standard error. Multiple statistical comparisons were calculated by ANOVA test, and the P value for each pairwise group was determined by Dunnett’s T3 (high variance between groups) or Bonferroni (low variance between groups) post-hoc test. The analysis of correlation was done using Pearson’s coefficient (r). Significance difference (p < 0.05) was depicted by asterisk.

Although OA was less harmful to HaCaT than BA (Fig. 1a), both significantly decreased the mitochondrial transmembrane inner potential (ΔΨm), as indicated by the incorporation of Rh123 and quantification of its fluorescence. Just 3 hours after the start of the treatment, BA and OA induced a significant decrease in Rh123 fluorescence of 46% and 16%, respectively p < 0.001 (Fig. 2a-i). Under the same experimental conditions, the mitochondrial uncoupling agent carbonyl cyanide m-chlorophenylhydrazone (CCCP)^24^ decreased ΔΨm by 18%, p = 0.003 (Fig. 2a-i). Despite the fact that both triterpenoids showed a significant decrease in Rh123 fluorescence, the toxic mitochondrial effects of BA was larger and better correlated with time (r = −0.7), than those induce by OA (r = −0.5) (Fig. 2a-ii).

By double staining HaCaT with MTR (a probe sensitive for ΔΨm) and LTG (lysosomotropic dye) after 6 hours of treatment, we were able to reproduce the loss of ΔΨm by all three drugs (BA, OA and CCCP). Note the decrease in MTR staining for BA, OA and CCCP compared with the control and the lysosome accumulation, especially in BA-treated cells (Fig. 2b). It is interesting that the cellular regions of BA-treated cells showing lysosome accumulation are the same that showed mitochondria ΔΨm loss (line scans in Fig. 2b). Merged images also revealed that lysosomes accumulated selectively on regions that had depolarized mitochondria, i.e., with less MTR fluorescence (Fig. 2b).

The degree of mitochondria sequestration by autophagosomes was analyzed by performing a triple staining protocol (COXIV, LTR, LC3-II) at T1. Compared with control, BA and OA-treated cells displayed an increase and decrease, respectively, in COXIV labeling (Fig. 2c). Both BA and OA-treated cells showed an increased level of LTR loaded lysosomes, which overlapped with mitochondria (COXIV) (Fig. 2c-i). Note also that the endogenous autophagosome marker LC3-II increased in both BA and OA-treated cells, indicating an activation of autophagy compared to control. It is interesting that in the case of OA autophagy activation caused a decrease in mitochondrial mass, suggesting efficient removal of damaged mitochondria, while in the case of BA there was significant accumulation of mitochondria (p = 0.004), suggesting failure in their autophagic removal (Fig. 2c-ii). Flow cytofluorometry analysis also supported the LC3-II lipidation upon treatment with BA parallel to mitochondrial mass accumulation (Fig. 2d-i). The appearance of a subpopulation of BA-treated cells (21.9%) presenting increase in both LC3-II and COXIV pointed to the significant accumulation of mitochondria inside autophagosomes (upper right quadrant) (Fig. 2d-ii). This subset of HaCaT was also observed for OA-treated cells, but with a moderate frequency (8.9%) when compared to the basal levels of the control (1.5%). On the other hand, a subset of OA-treated cells (21.8%) had an increase in LC3-II that was not correlated to the increase in mitochondrial mass as observed before in microscopy (Fig. 2c). Under BA the accumulation of mitochondria was so large that there was a significant population of cells (22.9%) with increased mitochondrial content without increase in LC3-II staining (Fig. 2d-i). We further analyzed HaCaT that had the autophagic flux inhibited by BAF (Fig. 2e). After gating mitochondria (COXIV) as function of LC3-II, cytofluorometric analysis revealed a subset of BA-treated cells (upper right quadrant) with high correlation between LC3-II and mitochondrial mass (80.1%) compared to control (26.3%) (Fig. 2e). Regarding inhibition of autophagy by BAF treatment, we did not observe significant change in this correlation (72.6%), which suggested that BAF *per se* was not capable of inhibiting autophagic removal of mitochondria.

In order to monitor the different steps of autophagic clearance in response to damaged mitochondria, we treated HaCaT with triterpenoids or CCCP and measured the activity of the enzyme citrate synthase (CS), which is located inside the mitochondrial matrix^25^. Within 6 hours of CCCP addition, a period of time sufficient for recruitment of lysosomes to depolarized mitochondria (Fig. 2b), we observed a significant decrease of citrate synthase (CS) activity (p = 0.024) (Fig. 2f), indicating effective mitophagy^24^. Using the same protocol the decrease in CS activity by OA (24%) was similar to that of CCCP, suggesting therefore effective mitophagy. Interestingly, HaCaT under BA showed almost no change in CS activity (10%) even after the impressive reduction of ΔΨm (2.5-fold) (Fig. 2a), indicating again that mitochondria clearance was not effective in this case.

Next, we evaluated the selective recruitment of the ubiquitin-ligase Parkin to damaged mitochondria, which is one of the initial steps of mitophagy activation^25^,^26^. Note that Parkin is dispersed in the cytosol of control cells and tend to accumulate in specific micro-environments in cells treated with BA, OA and CCCP (Fig. 2g). Note also that Parkin staining co-localized with COXIV in cells treated with BA, OA and CCCP but not in the control (line scans). Like CCCP, both triterpenoids induced the translocation of Parkin to depolarized mitochondria, indicating mitophagy activation. However, in the case of BA mitophagy seemed to be activated but ended up in mitochondrial accumulation instead of clearance, suggesting interruption of the autophagic flux. This result was compatible with the data presented in Fig. 2c, as well as with recent reports concerned with the biological effects of BA^20^.

### Unlike OA, BA compromises the autophagic flux

**Figure 3:**
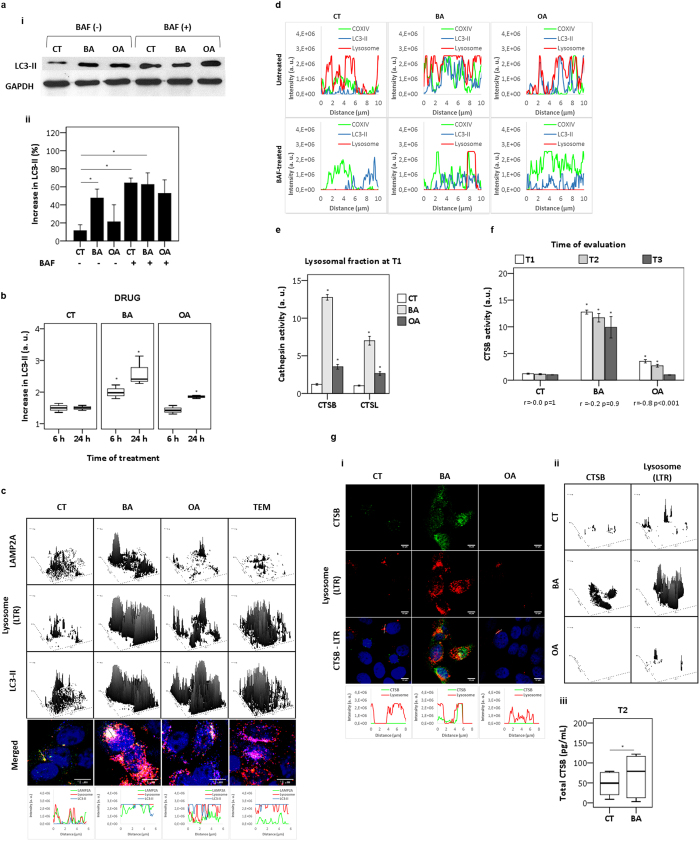
Consequences of autophagic flux inhibition. (**a**) LC3 lipidation (LC3-II form) after 6 hours of treatment with DMSO (control) and triterpenoids (20 μM) (**i**). Bars indicated the mean of relative amount of LC3-II (%) normalized by the baseline expression of GAPDH (**ii**). (**b**) Keratinocytes treated with DMSO (control) and triterpenoids (20 μM) were immunostained with LC3-II at indicated times (6 or 24 hours). Fold change of LC3-II compared to control were represented as arbitrary units. (**c**) At T1 following staining of lysosomes (LTR), cells treated with DMSO (control), triterpenoids (20 μM) or TEM (15 μM) were immunostained for LC3-II (blue) and for lysosomal marker LAMP2A (green). At bottom, plot profile (LC3-II/lysosome/LAMP2A) of lines drawn in the images. (**d**) At T1 HaCaT treated with DMSO (control) and triterpenoids (20 μM) in presence or absence of BAF (2 ηM). Following staining of lysosomes with LTR (red), cells were immunostained for LC3-II (blue) and for COXIV (green). Fluorescence plot profiles represented line scans of LTR-loaded lysosomes and LC3-II/COXIV. (**e**) At T1 lysosomal enzymes were extracted from subcellular fraction (lysosomes), following fluorescence assays for detection of cathepsins L (CTSL) and B (CTSB) activities. (**f**) CTSB activity from lysosomal fraction as a function of time. (**g**) At T2 HaCaT treated with DMSO (control) and triterpenoids (20 μM) following lysosomal staining with LTR (red) and immunostaining for total CTSB (green). Plot profile (CTSB/lysosome) of lines drawn in the images (**i**) and surface plots (**ii**). At T2 with DMSO (control) and BA (20 μM) total CTSB (ρg/mL) was assayed by ELISA (**iii**). T1 = after treatment for 24 hours; T2 = 24 hours after T1; T3 = 48 hours after T1. All results were obtained from at least three independent experiments and expressed as mean values ± standard error. Multiple statistical comparisons were calculated by ANOVA test, and the p-value for each pairwise group was determined by Dunnett’s T3 (high variance between groups) or Bonferroni (low variance between groups) post-hoc test. Significance difference (p < 0.05) was depicted by asterisk.

To evaluate further the autophagic flux inhibition by BA, we quantified LC3 lipidation^27^. The increase in lipidated LC3-II was found on western blots and by FACS even at 6 hours of treatment with BA (Fig. 3a,b). Note that for both control and OA-treated cells there was accumulation of the LC3-II form under inhibition of autophagic flux by BAF, which specifically inhibits the lysosomal proton pump (vacuolar H^+^ ATPase)^28^ (Fig. 3a-i). On the other hand, in the case of BA-treated cells the LC3-II form was significantly abundant (p = 0.040) regardless of autophagy inhibition by BAF (Fig. 3a-ii). Moreover, as observed by FACS, in BA-treated cells there was a significant accumulation of the LC3-II form (p < 0.001) in a time-dependent manner (Fig. 3b), indicating that BA inhibits the autophagic flux.

To test if BA compromised autophagosome fusion with lysosomes, we evaluated the co-localization between LC3-II, LAMP2A and lysosomes stained by LTR (Fig. 3c). After treatment with BA, OA and TEM for 6 hours, we observed an increase in LAMP2A expression mainly in BA-treated cells. As expected, parallel to the increase in endogenous LC3-II there was an enhancement of LTR loaded lysosomes (surface plots), which showed selective co-localization with LAMP2A (traced line). Plot profile analysis of LC3-II/LAMP2A and lysosomes indicated the appropriate formation of autolysosomes upon BA (Fig. 3c, line scans). Under OA there was a strong reduction in the level of COXIV and almost no co-localization with LTR and LC3-II, indicating that there was no accumulation of autolysosomes and suggesting again an effective removal of mitochondria, which is endorsed by lysosomal and LC3-II overlapping (lined area, Supplementary Figure S1)^29^. Under OA in the presence of BAF we saw a strong reduction in LTR staining and its loss of co-localization with LC3-II (Fig. 3d, line scans), showing that effective autophagy failed upon BAF treatment (Supplementary Figure S1). Under this experimental condition, OA-treated cells showed a remarkable increase in mitochondrial mass (p = 0.001), which is similar to that caused by BA without BAF (Supplementary Figure S1, surface and box-plots).

To further characterize the dysfunctional autophagy that seems to end up in cell death upon BA treatment, we quantified the activity of lysosomal fraction of cathepsins B and L (CTSB and CTSL, respectively). At T1, there was a significant increase in CTSB and CTSL for both BA and OA (Fig. 3e). In BA-treated cells, we observed a remarkable increase in CTSB and CTSL activities of 13 and 7-fold compared to control, respectively. Although at lower level, OA enhanced CTSB and CTSL activities to 4 and 3-fold compared to control, respectively (Fig. 3e). This enhanced activity of CTSB in OA-treated cells decreased with time, while under BA the increase was sustained regardless of time (Fig. 3f). The increase of CTSB co-localized with lysosomes at T2 was clearly observed after the treatment with BA and not with OA (Fig. 3g-i). Note that the increase in CTSB at the lysosomal fraction (Fig. 3g-ii) was so large that it could even be detected by ELISA, p = 0.023 (Fig. 3g-iii). These findings support a scenario of BA mediating lysosome and autophagolysosome accumulation, which are, however, non-functional.

### BA destabilizes lysosomes

**Figure 4:**
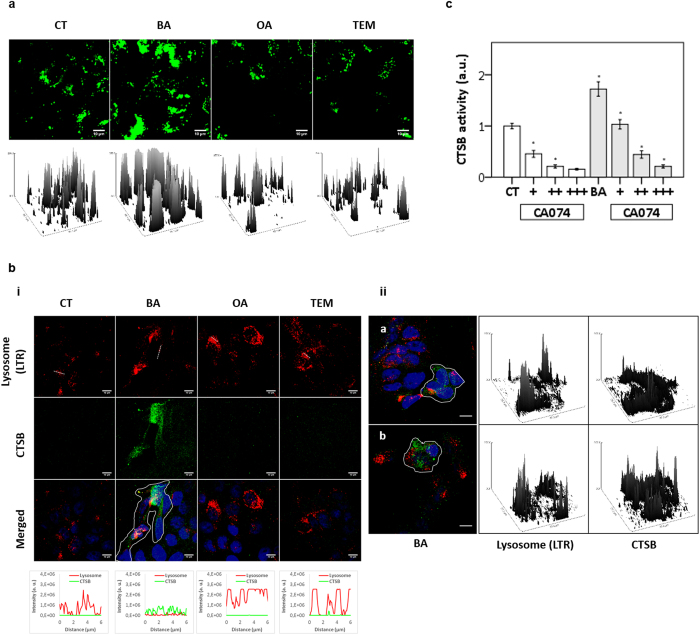
BA desastibilized lysosomes of HaCaT keratinocytes. (**a**) After treatment for 6 hours with DMSO (control), triterpenoids (20 μM) or TEM (15 μM) cells were stained with LTG and evaluated under confocal microscope. Surface plots below the images represented the fluorescence of LTG-loaded lysosomes. (**b**) Within 6 hours of DMSO (control) and triterpenoids (20 μM) addition, cells were stained with LTR (red) and immunostained for total CTSB (green). (**i**) Fluorescence plot profiles represented line scans of LTR-loaded lysosomes and CTSB (bottom). Micrographs of BA-treated cells (lined area) showing elevated CTSB in cytosol associated with decrease in LTR-loaded lysosomes (**ii**). At right, surface plots of depicted area drawn in the images (**ii**). (**c**) Cathepsin B (CTSB) activity from cytosolic fraction within 3 h of BA (20 μM) addition upon absence (–) or presence of CTSB inhibitor CA074 at 5 μM (+) and 10 μM (++). T1 = after treatment for 24 hours; T2 = 24 hours after T1; T3 = 48 h after T1. All results were obtained from at least three independent experiments and expressed as mean values ± standard error. Multiple statistical comparisons were calculated by ANOVA test, and the p-value for each pairwise group was determined by Dunnett’s T3 (high variance between groups) or Bonferroni (low variance between groups) post-hoc test. Significance difference (p < 0.05) was depicted by asterisk.

Micrograph analysis of live cells revealed cytoplasmic staining with the lysosomotropic LTG for all experimental conditions – control, BA, OA and TEM (Fig. 4a). Six hours after treatment with BA (but not with TEM or OA) cells revealed accumulation of lysosomes displaying higher LTG-loads (Fig. 4a, surface plots). To further evaluate the effects of BA on lysosomes we investigated lysosomal membrane permeabilization (LMP), which was measured in terms of the release of CSTB to cytosol^30^.

The loss of lysosomal membrane integrity was measured in terms of decrease in LTR-loading by lysosomes^30^,^31^ (Fig. 4b). Within 6 hours of treatment with BA, unlike control and OA, we found a subset of cells (lined area) with relevant decrease in the lysosomal LTR-loading (Fig. 4b-i). Parallel to lysosomal membrane instability we observed cytosolic increase in CTSB only after BA treatment (Fig. 4b-ii), which was observed neither under OA nor TEM (Fig. 4b-i, line scans).

To quantify the cytosolic CTSB (CTSB protease activity in cytosolic extracts) 6 hours after BA treatment, we analyzed the ability of cleaving the synthetic substrate RR-AFC to release free AFC. Under BA, there was a significant increase in the cytosolic activity of CTSB (Fig. 4c). This activity significantly and specifically decreased (p < 0.001) under presence of CA074, a selective inhibitor of CTSB^21^.

Since accumulation of non-functional autolysosomes and LMP are intrinsically associated with cell death^11^, we investigated their relation with apoptosis. Under BA there was a significant increase in plasma membrane permeabilization (PI positive cells) as revealed by cytofluorometric analysis (Supplementary Figure S2b). The proportion of apoptotic cells, characterized by the presence of negatively charged lipids that bind to Annexin V (AV) without losing cytoplasmic membrane integrity and therefore avoiding cellular incorporation of Propidium Iodide (PI), were only slightly increased under BA. The percentage of (AV^+^/PI^−^) cells for BA was 2% compared to control (p = 0.32). Cells with positive AV staining parallel to cellular incorporation of PI (AV^+^/PI^+^) showed a significant increase after BA treatment (14%) compared to control (6%) (p = 0.043). In the case of OA treatment, there was no significant difference for both AV^+^/PI^−^ and AV^+^/PI^+^ populations compared to control. The proportion of AV^−^/PI^+^ cells was higher under BA (24%) compared to control (12%) or OA (11%) (p-values of 0.024 and 0.015, respectively, Supplementary Figure S2b). At T1, we also checked for activation of caspase-3, a major modulator of apoptosis, by FACS (Supplementary Figure S2c). Only BA induced an increase in the level of cleaved caspase-3. Accordingly, after gating HaCaT as a function of cell size (FSC) and caspase-3 staining, we revealed that a minor population of treated cells with BA (3.4%) and OA (0.4%) showed increase in caspase-3 compared to control (0.2%) (Supplementary Figure S2g). Interestingly, among caspase-3 positive cells we found a major subpopulation (upper right quadrant) with increase in cell size. This cell enlargement agreed to data showed before for BA (Fig. 1f). In summary, after BA treatment a small fraction of cells showed apoptotic response. However, this small reaction did not correspond to the large loss of ΔΨm and important LMP caused by BA (Figs 2a, 4b, c). In fact, as shown above the large majority of cells die in response to the parallel damage in mitochondrial and lysosomal membranes, leading to mitochondrial-lysosomal axis of cellular stress.

### Triterpenoids induce damage in membrane-based models

The data showed above brings an important question: why is BA more efficient than OA in destabilizing lysosomes? In order to answer it, we analyzed the ability of these triterpenoids to interact with membranes both *in vitro* and *in silico*.

**Figure 5:**
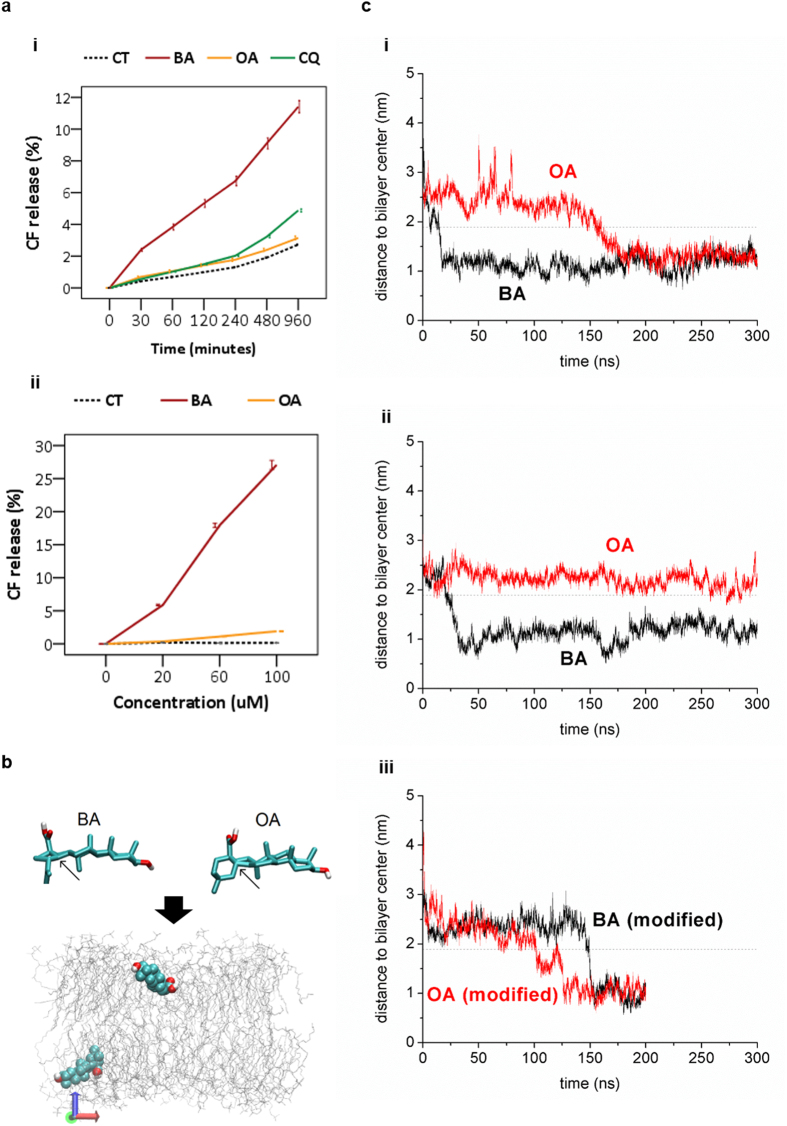
Ligand-interactions of triterpenoids in mimetic membranes. (**a**) The analysis of cell membrane permeability was based on the release of carboxyfluorescein - CF. The percentage of CF-release was calculated in a time (**i**) and dose-dependent manner (**ii**). (**b**) DPOC, di-oleoyl-phosphocholine (C18∶1) bilayer was submitted to dynamic analysis of membrane interaction. Chemical structures of BA and OA (upper panel). Color coding: C (cyan), O (red) and polar H (white). The arrows indicate the chirality centers relevant for (**C**-**i**). Snapshot of the simulated system after 300 ns showing both BA and OA (van der Walls spheres) and the phospholipid bilayer depicted in gray. (**c**) Time evolution of the distance between triterpenoids and the bilayer center (**i**); in a second independent run (**ii**); and when the structure of both BA and OA was modified (**iii**) by inverting the chirality of the carbon atoms indicated by arrows in (B, upper triterpenoids structures). The dashed line represents the bilayer headgroups region. All results were obtained from at least three independent experiments and expressed as mean values ± standard error.

Despite the structural and physicochemical similarities between OA and BA (Table 1), BA exhibited a much stronger ability to interact and damage synthetic membrane. Initially, we evaluated the disruption of liposomes in which the release of internal material (carboxyfluorescein) was measured as a function of time and BA concentration. Accordingly, BA disturbed the liposome system in a dose and time-dependent manner (Fig. 5a). Contrarily, OA and CQ were not capable of interacting directly with liposomes (Fig. 5a). Although CQ impaired lysosomal function^10^,^32^ mostly due to enlargement of lysosomes by proton sponge effect^30^,^33^, it did not induce direct damage to the mitochondrial membrane as BA did (data not shown). At equal concentrations (20 μM), BA was much more cytotoxic than CQ and OA in HaCaT^10^. These observations indicate that the disturbance of the membrane structure may be the basis of BA toxicity.

In spite of being chemical isomers, BA and OA have subtle structural differences. BA has a planar backbone, while in OA there has a twist between the two rings connected to the carboxyl group (Fig. 5b). Molecular dynamics simulations showed that both BA and OA were able to penetrate into a POPC, 2-oleoyl-1-palmitoyl-sn-glycero-3-phosphocholine (C16,18:1) bilayer down to the region immediately bellow the lipid headgroups (Fig. 5b), but with different efficiencies and kinetics. In a first simulation, both triterpenoids reached a distance of ca. 1.3 nm from the bilayer center and remained at this region until the end of the simulation (300 ns). However, the speed of the immersion process was considerably faster for BA. It took ca. 20 ns for BA to reach its equilibrium position bellow the phosphate headgroups, while the same process took ca. 170 ns for OA. We performed an additional simulation with the drugs initially placed at different positions. In this second independent run an immersion time of 25 ns was obtained for BA, while OA did not penetrate at all during the full 300 ns run (Fig. 5c). Next, we performed additional simulations in which one of the carbon atoms at the backbone end (indicated by an arrow in Fig. 5b) had its chirality inverted. This led to a twisted structure in BA and a fully planar structure in OA. As shown in Fig. 5c-iii, the immersion of BA was significantly delayed upon chirality inversion. In a similar manner, the time required for OA penetration was reduced. This is a clear indication of the influence of the backbone twist on the speed of penetration of cyclic triterpenoids in model membranes.

### OA behaves similarly to BA when lysosomes is chemically impaired

**Figure 6:**
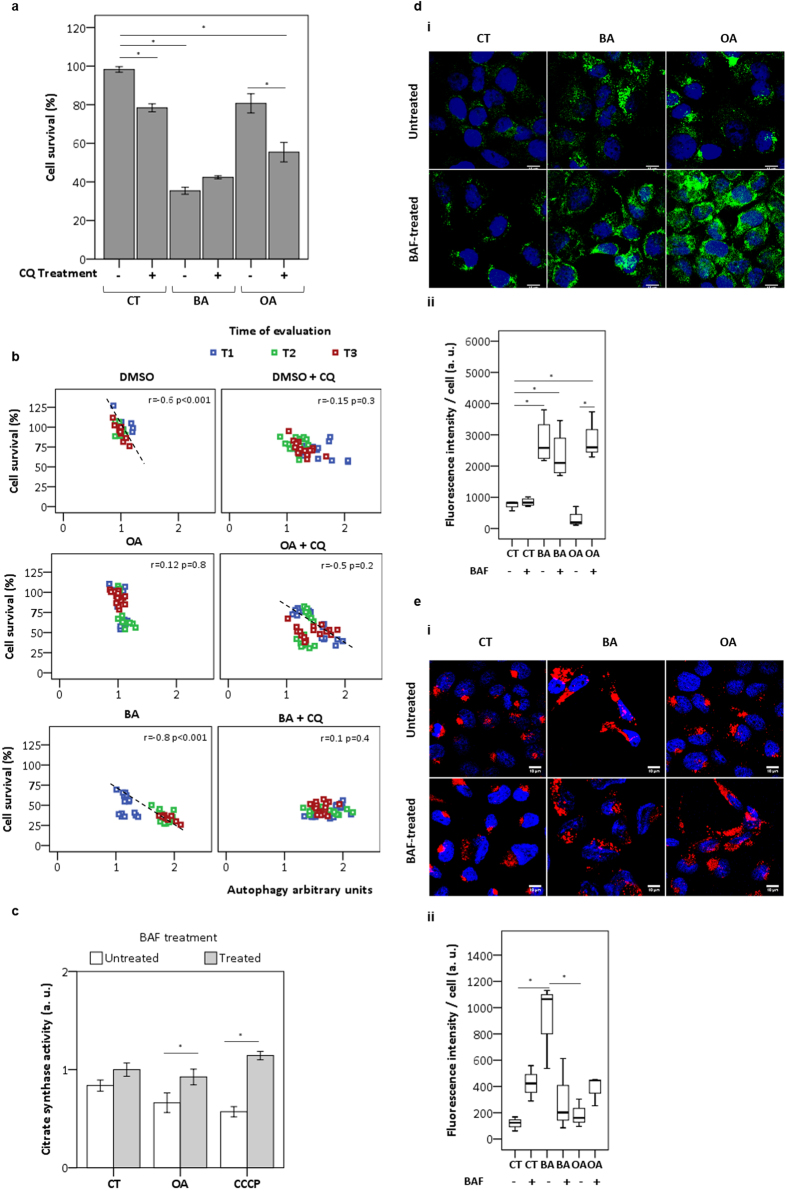
OA became cytotoxic after lysosomal impairment. (**a**) HaCaT keratinocytes were treated with DMSO (control) and CQ (25 μM) for 24 hours. (**b**) At indicative times cell survival was evaluated by MTT assay following calculation of AAU. The correlation between cell survival and AAU was evaluated as function of time T1, T2 and T3. (**c**) After treatment for 6 hours with DMSO (control), OA (20 μM) or CCCP (2 μM) in presence or absence of BAF (2 ηM) citrate synthase activity was presented as arbitrary units after normalization to control. Micrographs of treated cells at T1 with DMSO (control) and triterpenoids (20 μM) in presence or absence of BAF (2 ηM) treatment, following immunostaining for (**d**) the mitochondrial marker COXIV (green) and (**e**) p62 (red). At bottom panel, box-plots represented the fluorescence profile of multiple images. T1 = after treatment for 24 hours; T2 = 24 hours after T1; T3 = 48 h after T1. All results were obtained from at least three independent experiments and expressed as mean values ± standard error. Multiple statistical comparisons were calculated by ANOVA test, and the p-value for each pairwise group was determined by Dunnett’s T3 (high variance between groups) or Bonferroni (low variance between groups) post-hoc test. The analysis of correlation was done using Pearson’s coefficient (r). Significance difference (p < 0.05) was depicted by asterisk.

Although efficient autophagy avoided the accumulation of OA-damaged mitochondria in HaCaT, we asked whether OA would trigger cell death similarly to BA if an extrinsic lysosomal damage was induced. For that, we investigated the effects of OA on HaCaT cellular survival after inhibiting lysosome activity with CQ or BAF (Fig. 6a). As expected, at T2 the cellular survival rates observed for BA-treated cells did not significantly change regardless of the presence of CQ, p = 1.0 (Fig. 6a). Contrarily, OA-treated cells in the presence of CQ exhibited significant decrease in cell survival (55%) compared to control (80%), p < 0.001. Next, to test if this decrease of cellular survival was significantly correlated with accumulation of lysosomes, as quantified by AAU^10^, we evaluated cell response as a function of time (Fig. 6b). OA-treated cells showed decrease of cell survival correlated to increase in AAU upon inhibition of autophagy by CQ (r = −0.5). On the other hand, in the case of BA we observed a lack of correlation under autophagy inhibition by CQ (r = 0.1) compared to control BA (r = −0.8).

The inhibition of autolysosome content degradation triggered by BAF in combination with OA or CCCP caused a significant increase in CS activity when compared to BAF untreated cells (Fig. 6c). This increase can be attributed to the huge accumulation of mitochondrial mass, as shown by fluorescence microscopy (bulk green) (Fig. 6d-i). Unlike OA, BA-treated cells did not show remarkable alteration of mitochondrial mass when treated with BAF (Fig. 6d-ii). In an attempt to further examine the impact of OA treatment in cells with defective lysosomes induced by BAF, we evaluated the recruitment of adaptor molecules such as p62, which represents a general selective degradation signal in mammalian cells, not only for protein aggregates but also for membrane-bound organelles^27^. Herein, the labeling of the endogenous p62 in the cytosol of OA-treated cells increased upon lysosomal impairment (Fig. 6e-i). Note that in the case of BA there was a decrease of p62 upon autophagy inhibition by BAF (Fig. 6e-ii). Other data in the manuscript concerning damage in lysosome or mitochondria by BA also indicated that OA behave similarly to BA if lysosomal function is somehow compromised (Supplementary Figure S1, Fig. 3a,d). Therefore, if lysosome is damaged, OA also induces efficient cell death with autophagy (Fig. 6b).

## Discussion

BA is a promising anti-cancer agent and the entire family of pentacyclic triterpenoids may provide new leads for anti-cancer and anti-microbial drugs^34^,^35^. Both BA and OA caused significant depolarization of mitochondrial membrane, but only BA efficiently induced cell death. Although we observed small fractions of cells with cytoplasmic membrane permeabilization (AV^−^/PI^+^) and with apoptotic phenotype (AV^+^/PI^−^, AV^+^/PI^+^ and caspase 3 activation), neither necrosis nor apoptosis were the main mechanisms of cell death. The few dying cells showing these characteristics also showed conspicuous vacuolization (granularity), parallel to enhancement of mitochondrial mass. The proportion of dead cells increased with lysosomal instability in a time-dependent manner, indicating cell death with autophagy. As demonstrated for several other cell types^18^,^20^, we have shown that BA also activates autophagy in HaCaT and HeLa cells.

OA treatment resulted in marked cytoplasmic vacuolization and mitochondrion shrinkage with remarkable cellular recovery that was intrinsically associated with autophagy activation. However, cell recovery failed upon treatment with lysosomal inhibitors (Chloroquine or Bafilomycin-A1). As previously shown^20^, autophagic flux was found to be non-functional after BA treatment, leading to diminished degradation of long-lived LC3-II and p62 proteins. The lysosomal damage BA-mediated was *per se* capable of compromising autophagy, without any incremental damage when lysosomal function was deeply altered by lysosomal inhibitors (CQ or BAF). BA-vacuolated cells survived for several hours and showed a remarkable mitochondrial accumulation. This later event probably led to continuous loss of lysosomal function, turning autophagy into a destructive process^36^. The relationship between cell death and acid vacuole accumulation was quantified by the correlation between AAU^10^ and cell death.

BA disrupted lysosome membrane causing some leakage of cathepsins; however, lysosomal leakage was not the main effect of BA, since most lysosomes were able to maintain pH gradient (as shown by the accumulation of lysosomotropic dyes). In addition, over time, there is a strong accumulation of cathepsins within lysosomes. The damage in lysosomal function caused by BA cannot either be explained by traditional mechanisms such as lack of lysosome acidification or neutralization, since BA and AO have almost identical acid-base equilibria (Table 1). The main differences between the biological effects of BA and OA were due to their efficiencies in interacting with and damaging membranes. BA is miscible with the outer leaflet membrane phospholipids^37^, being capable of changing permeability of mimetic membranes^38^,^39^. In this study, unlike OA, BA induced significant *in vitro* membrane leakage that was associated with *in vivo* membrane impairment of mitochondria and lysosome. The ability of BA to disturb the mitochondrial membrane is in agreement with other data that shows specific interactions between BA and Cardiolipin in Langmuir monolayers^38^. BA was also shown to disrupt membranes of human red blood cells (RBC) *in vitro*^39^.

**Figure 7:**
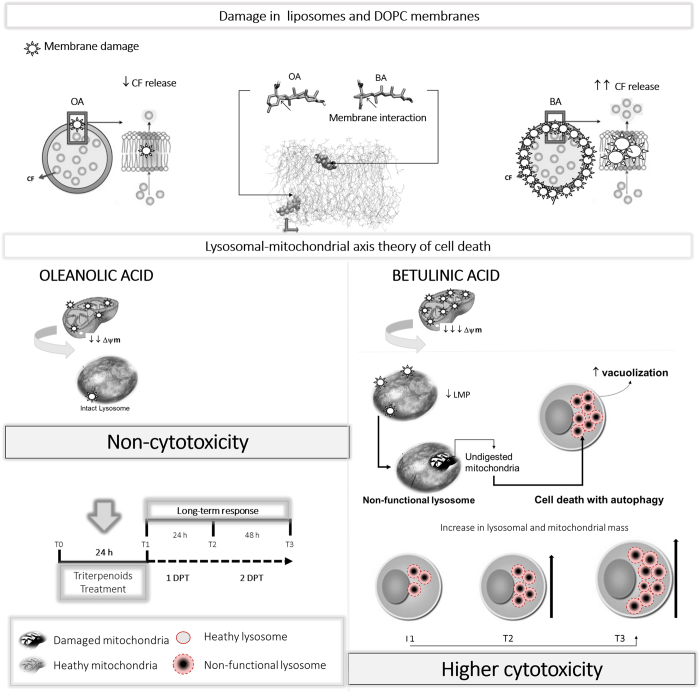
Scheme of triterpenoids effects on HaCaT keratinocytes. Parallel damage in the mitochondrial and lysosomal membranes leads to cell death with autophagy. This figure was drawn by W. K. M.

Taken together, our results indicate a new paradigm concerning the fate of autophagy. Whether it will cause cell rescue or cell death, it seems to depend on the extent of membrane damage, as summarized in Fig. 7. The late harmful effects of BA relates to a mitochondrial-lysosomal axis of cellular stress, which will end up causing cell death with autophagy in HaCaT. This paradigm explains the higher cytotoxicity of BA compared to OA and may help in the search for new anti-tumoral molecules^40^.

Since many drugs that suppress autophagy flux act as weak bases, by studying BA and OA we proposed an alternative mechanism to compromise autophagy based on disturbance of the membranes of key organelles. Our findings suggest that causing early functional activation of autophagy due to mitochondrial damage parallel to higher impairment of lysosomes may represent an interesting strategy to attack multi-drug resistant tumors that escape apoptosis^41^.

It is interesting to consider that BA targets membranes of organelles (mitochondria and lysosome) but seems to preserve the cytoplasmic membranes. It is very likely that BA molecules enter cells by endocytosis and therefore the molecular answer of why cytoplasmic membrane is not affected by BA will have to await a more complete understanding of the intracellular membrane trafficking and on the effects of BA. We hope this paper contributes to future work on this direction.

## Methods

### Cell lines and cell culture

We cultured immortalized human skin keratinocytes (HaCaT)^8^ and epidermoid carcinoma of the cervix (HeLa)^42^ in Dulbecco’s modified Eagle’s medium (DMEM, Sigma-aldrich) supplemented with 10% (v/v) fetal bovine serum (FBS), 100 U/mL of penicillin, 25 μg/mL of amphotericin B and 100 μg/mL of streptomycin in a 37 °C incubator at a moist atmosphere of 5% carbon dioxide. HaCaT cell lines were gently supplied by Dr. Hugo Armelin, Butantan Institute, Brazil. HeLa cell lines were gently supplied by Dra. Mary Sogayar, NUCEL, Brazil.

### Correlation of cell death with lysosome accumulation

Triterpenoids BA and OA (Sigma-aldrich) were dissolved in DMSO (4 mg/mL) and diluted to the required concentration in DMEM (Sigma-aldrich) supplemented with 1% (v/v) fetal calf serum (FBS), 100 U/mL of penicillin, 25 μg/mL of amphotericin B and 100 μg/mL of streptomycin in a 37 °C incubator at a moist atmosphere of 5% carbon dioxide. After seeding, we treated exponentially growing human HaCaT with triterpenoids at increasing concentrations (10–30 μM), and incubated for 24 hours at 37 °C. Next, to quantify the augmentation of lysosomal compartment intrinsically correlated to cell death we applied a recent strategy based on a numeric variable AAU (autophagic arbitrary units)^10^. Briefly, its conceptual framework is the uptake of the lysosomotropic reagent Neutral Red (Sigma-aldrich) in enlarged autophagic vacuoles engaging in cell death. This NR-uptake was then weighted by the average of cell survival measured by MTT and CVS (Crystal Violet Staining) assays, allowing the calculation of AAU. For this, cells were stained with 30 μg/mL of NR at 37 °C for 2 hours. After washing, NR was eluted with an alcoholic-based 1% (v/v) acetic acid fixing solution and measured at 540 nm, using the microplate reader Infinite^®^ 200 PRO (TECAN). These fixed cells after washing with water were used for CVS assay.

### Cellular cytotoxicity

To analyze survival rate rather than short-term cytotoxicity, we allowed HaCaT to proliferate for two population doubling times (PDTs) after treatment with BA or OA (both from Sigma-aldrich) in a dose-dependent manner (10–30 μM) for 24 hours at 37 °C. We performed independently CVS and MTT assays. Briefly, after treatment we added cells to medium containing MTT (Sigma-aldrich) at 50 μg/mL and incubated for 2 hours at 37 °C. Next, the MTT reduced-product formazan was solubilized in DMSO (Sigma-aldrich), and absorbance values were read at 550 nm, using the microplate reader Infinite^®^ 200 PRO (TECAN). For the CVS assay, fixed cells from NRU-assay were stained with Crystal Violet (CV, Sigma-aldrich) at 0.02% (w/v) for 5 minutes at room temperature. After washing, we eluted CV with 0.1 M sodium citrate in 50% (v/v) ethanol, and recorded absorbance values at 585 nm^10^.

### Immunostaining (microscopy and FACS)

For comparative analysis, HaCaT keratinocytes were treated with triterpenoids (20 μM) for 24 hours, and the biological effects were observed after indicative times. After fixation and blocking, we incubated slides with primary rabbit monoclonal antibodies against caspase-3 active form (CASP3, Cell Signaling Technology), microtubule-associated proteins 1B (LC3B, Cell Signaling Technology), ubiquitin binding protein sequestosome 1 (SQSTM1/p62, Cell Signaling Technology) and the heavy chain of mature Cathepsin B (CTSB, Abcam). We used also primary mouse monoclonal antibodies against lysosome-associated membrane protein 2 (LAMP2A, Abcam) and cytochrome c oxidase subunit IV (mouse IGg COXIV, Molecular Probes) according to manufacturer’s instructions. To reveal the primary antibody linkage we used goat antibodies against mouse IgG (Alexa 488 or 633) or rabbit IgG (Alexa 488 or 633), all from Molecular probes (Eugene, OR, USA). We analyzed 4,6-diamidino-2-phenylindole (DAPI) counterstained slides under confocal microscope (Zeiss™ Axiovert 200 LSM 510 Laser and Confocor Modules) equipped with a Plan-APOCHROMAT 63X/1.40 oil DIC M27 objective (Zeiss™) and imaged using ImageJ Software (National Institutes of Health). Alternatively, we measured CASP3, LC3-II and COXIV related-fluorescence using flow cytometry (BD FACS Verse^™^). At least 20,000 events were collected in each analysis. Data was further analyzed by FlowJo software. Following guidelines^27^, we washed treated-cells with PBS containing digitonin (0.25 mg/mL) and processed for FACS analysis for endogenous LC3-II.

### Labeling of acidic compartments in live cells

For comparative analysis, HaCaT keratinocytes were treated with triterpenoids (20 μM), and the acidic compartments were evaluated after indicative times. Both lysosomotropic dyes Acridine Orange (AO, Sigma-aldrich) and acidophilic lysosomal probes LysoTracker Green (LTG, Molecular Probes) and LysoTracker Red (LTR, Molecular probes) were used as probes to primarily detect lysosomes^11^,^27^. In brief, we incubated HaCaT with 100 ηM LTG, 500 ηM LTR or 1 μg/mL AO in DMEM 1% (v/v) FBS at 37 °C for 15 min. After washing, we immediately analyzed live-cells under confocal microscope as described above. Since LTR-loading remained retained in lysosomes even after aldehyde fixation, we could performed confocal microscopy following immunostaining. Alternatively, we collected at least 20,000 events to further flow cytofluorometric analysis of AO (BD FACS Verse^™^) using FlowJo software.

### Mitochondrial function

HaCaT keratinocytes were treated with triterpenoids (20 μM), and the mitochondrial function was evaluated after indicative times. According to manufacturer’s instructions the following fluorescent probes were used to examine mitochondrial function^43^: Rhodamine 123 (Rh123, Sigma-aldrich) and MitoTracker Red CMH_2_XRos (MTR, Molecular Probes) to monitor mitochondrial inner transmembrane potential (ΔΨm); MitoTracker Green FM (MTG, Molecular Probes) to measure cellular mitochondria content. MTG is a cell-permeant mitochondrial-specific dye that becomes fluorescent only on sequestration by mitochondria^44^. However, unlike RH123 and CMH_2_XRos, MTG covalently binds to mitochondrial proteins and thus can be used as a measure of mitochondrial mass independent of ΔΨm^44^. To quantify the fluorescence emission of MTG or Rh123 we collected at least 30,000 events to further cytofluorometric analysis (BD FACS Verse^™^). To analyze the data we used the FlowJo software. Alternatively, we used confocal microscopy to monitor ΔΨm regarding MTR fluorescence.

### Annexin V-FITC/PI double-labeled flow cytometry

The apoptosis phenotype was measured in HaCaT treated with triterpenoids (20 μM) at T1. Two-color flow cytometry was applied to detect the expression of Annexin V-FITC and the exclusion of PI. The cells positive for Annexin V-FITC and negative for PI represented the early apoptotic cells, whereas the cells positive for both markers represented the late apoptotic cells. The total apoptosis (%) was the sum of the early and late apoptotic cells. Briefly, after the treatment HaCaT keratinocytes were collected and washed twice with PBS and resuspended in 500 μL binding buffer. A total of 5 μL of Annexin V-FITC (Sigma-Aldrich) and 5 μL of PI were added, and the samples were maintained at room temperature for 10 min in the dark. To quantify the fluorescence emission of Annexin V-FITC (FL1) or PI (FL3) we collected at least 20,000 events to further cytofluorometric analysis (BD FACS Verse^™^). To analyze the data we used the FlowJo software.

### Western blot

Cell lysates were prepared in lysis buffer [20 mM PIPES, 100 mM NaCl, 1 mM EDTA, 10% (w/v) sacarose, 0.1% (v/v) CHAPS, 0.1% (v/v) Triton X-100, 1 mM PMSF, 2 μM Pepstatin A, 50 μM digitonin]. 20 μg of total proteins were separated in 12% acrylamide gels and transferred to nitrocellulose membranes (GE healthcare) using a semidry system (GE healthcare). Membranes were incubated in PBS with 5% (v/v) milk and 0.1% (v/v) Tween 20 for one hour and with primary antibodies (anti-LC3 from Cell Signaling Technology and anti-GAPDH from Sigma) in PBS with 2.5% (v/v) milk and 0.1% (v/v) Tween 20 for two hours. Three washing steps with PBS 1 × 0.1% (v/v) Tween 20 for 10 min were performed and followed by incubation with secondary antibody (anti-rabbit HRP from KPL) diluted in PBS with 2.5% (v/v) milk and 0.1% (v/v) Tween for one hour. Membranes were washed twice in PBS 1 × 0.1% (v/v) Tween 20 and once in PBS, 10 min each. After incubation with ECL Prime Western Blotting Detection Reagent (GE healthcare) for five minutes, membranes were exposed to X-Ray films. Images (in TIF files) were analyzed using ImageJ software and the results were normalized to GAPDH band intensities.

### Citrate synthase assay

Mitochondrial content over time was measured by assaying the citrate synthase activity, which decreases linearly regard with autophagy induction^27^. Following treatment with 2 μM CCCP (Sigma-aldrich) and 20 μM triterpenoids for 6 hours at 37 °C, HaCaT keratinocytes were washed once with PBS and lysed on the plate in 0.25% (v/v) Triton X-100 in PBS supplemented with protease and phosphatase inhibitors. Debris was removed by centrifugation and CS activity measured by following the oxidation of 5,5′-Dithio-bis(2-nitrobenzoic acid) in a spectrophotometer (absorbance at 412 nm) over time at 37 °C in the presence of acetyl co-enzyme A and oxaloacetate. Protein concentration in the same aliquot was measured using the BRADFORD assay (Bio-Rad Laboratories, Hercules, CA, USA) and enzyme activity normalized by total protein and expressed as arbitrary units (a. u.) after ratio to control (DMSO).

### CF-released measurement

To analyze the ability of triterpenoids to interact with membranes we performed a membrane-based mimic of cell membranes, which is based on the release of carboxyfluorescein (CF, Sigma-aldrich) from unilamellar liposomes, as previously described^45^. Liposomes were treated with Chloroquine IC_50_ (60 μM) and triterpenoids (20 μM) after the indicated times, or with triterpenoids in a dose-dependent manner (20 to 100 μM) for 6 hours. We calculated the rates of CF leakage as percent of total trapped CF release (Ft) under 0.2% (v/v) Triton X-100.

### Measurement of Cathepsin activities

HaCaT keratinocytes were treated with triterpenoids (20 μM) for the indicated time and the activity of cathepsins was evaluated by a fluorimetric assay (BioVision Inc.). Briefly, HaCaT keratinocytes were lysed in 50–100 μL of chilled lysis buffer. After 10 min incubation on ice, cytosolic fraction was separated from the rest of the cell debris by centrifugation at 10,000 g at 4°C for 5 min and supernatant was retained for analysis of cytosolic cathepsins related to LMP. Alternatively, whole-cell extracts were obtained after lysing the cell debris above with more 50–100 μL of chilled lysis buffer followed by freezing/thawing cycles (3×). Next, these whole-cell lysates were centrifuged at 10,000 g for 10 min at 4 °C, and 15 μg of protein per sample was used for enzymatic assays Protein concentration in the same aliquot was measured using the BRADFORD assay (Bio-Rad Laboratories, Hercules, CA, USA) and enzyme activity normalized by total protein and expressed as arbitrary units (a. u.) after ratio to control (DMSO). The quantification of lysosome cathepsin was performed by subtracting from the total cathepsin the cytosolic cathepsin activity.

### Measurement of total cathepsin B by ELISE

HaCaT keratinocytes were treated with BA (20 μM) for the indicated time and the quantity of total cathepsin B (CTSB) was evaluated by ELISA assay (R&D Systems). Briefly, whole-cell extracts were obtained as described above. Next, these whole-cell lysates were centrifuged at 10,000 g for 10 min at 4 °C, and at least 15 μg of protein per sample was used. Protein concentration in the same aliquot was measured using the BRADFORD assay (Bio-Rad Laboratories, Hercules, CA, USA) and total CTSB normalized by total protein and expressed as ρg/mL.

### Molecular dynamics simulations on DOPC bilayers

Molecular dynamics simulations were performed using the GROMACS 4.5.1 simulation package^46^,^47^. Molecular motions were computed by numerical integration of Newton’s equations with a time step of 2 fs. Fully hydrated lipid bilayers of POPC were represented using interaction parameters that have been previously validated against experimental membrane properties^48^. For compatibility, the pentacyclic triterpenoids BA and OA were assembled using the standard functional groups in the GROMOS53A6 force field^49^. Starting configurations were obtained from a pre-equilibrated membrane patch with 128 lipid molecules. One triterpenoid per membrane leaflet was initially placed at the aqueous phase at a distance of ca 3 nm from the bilayer surface. Overall, each simulated system had lateral dimensions of ca. 6.2 nm parallel to the membrane surface (*xy*-plane) and ca. 8.5 nm along the bilayer normal (*z*-axis). Periodic boundary conditions were applied in all Cartesian directions. The simulation protocol started with an equilibration run for 5.5 ns, during which the position of all triterpenoids was kept restrained. These molecules were then released and molecular trajectories were recorded for 300 ns under controlled temperature (310 K) and pressure (1 atm).

### Statistics

Statistical analysis was performed using IBM^®^ SPSS Statistics version 20. We analyzed data obtained from at least three independent experiments (n = 3), and expressed as mean values ± standard error. To perform comparative statistical analysis, we first analyzed the variance between groups. Next, multiple comparisons were performed using one-way analysis of variance (ANOVA) with Dunnett’s T3 or Bonferroni post-hoc test, depending on homogeneity of variance. The analysis of correlation was done using Pearson’s coefficient (r). An α = 5% (p < 0.05) was considered in every case to be statistically significant.

## Additional Information

**How to cite this article**: Martins, W. K. *et al.* Parallel damage in mitochondrial and lysosomal compartments promotes efficient cell death with autophagy: The case of the pentacyclic triterpenoids. *Sci. Rep.* **5**, 12425; doi: 10.1038/srep12425 (2015).

## Supplementary Material

Supplementary Information

## Figures and Tables

**Table 1:** Physicochemical properties of triterpenoids BA and OA.

									

Source: PubChem Compound [http://pubchem.ncbi.nlm.nih.gov/].
