# Supplementary material for: Parallel damage in mitochondrial and lysosomal compartments promotes efficient cell death with autophagy: The case of the pentacyclic triterpenoids
Source: Sci Rep. 2015 Jul 27;5:12425. doi: 10.1038/srep12425 (PMC4515638; doi:10.1038/srep12425)
Supplement: Supplementary Information [file srep12425-s1.pdf]

Parallel damage in mitochondrial and lysosomal compartments promotes efficient cell death with autophagy: The case of the pentacyclic triterpenoids

Waleska K. Martins[1], Érico T. Costa[2], Mário C. Cruz[3]; Beatriz S. Stolf[3], Ronei Miotto[4], Rodrigo M. Cordeiro[4], Maurício S. Baptista[1,*]

[1]Instituto de Química, Universidade de São Paulo, Brazil; [2]Ludwig Institute for Cancer Research (LICR) at Centro de Oncologia Molecular, Hospital Sírio Libanês, São Paulo, Brazil; [3]Instituto de Ciências Biomédicas, Universidade de São Paulo, Brazil; [4]Centro de Ciencias Naturais e Humanas, UF-ABC, Brazil.

To whom the correspondence should be addressed: Maurício S. Baptista; Address: Department of Biochemistry, Sao Paulo University, Av. Prof. Lineu Prestes 748 room 1262. CEP 05508-000 Sao Paulo, Brazil. Phone: (55) 11 30918952; FAX: (55) 11 38155579; E-mail: baptista@iq.usp.br

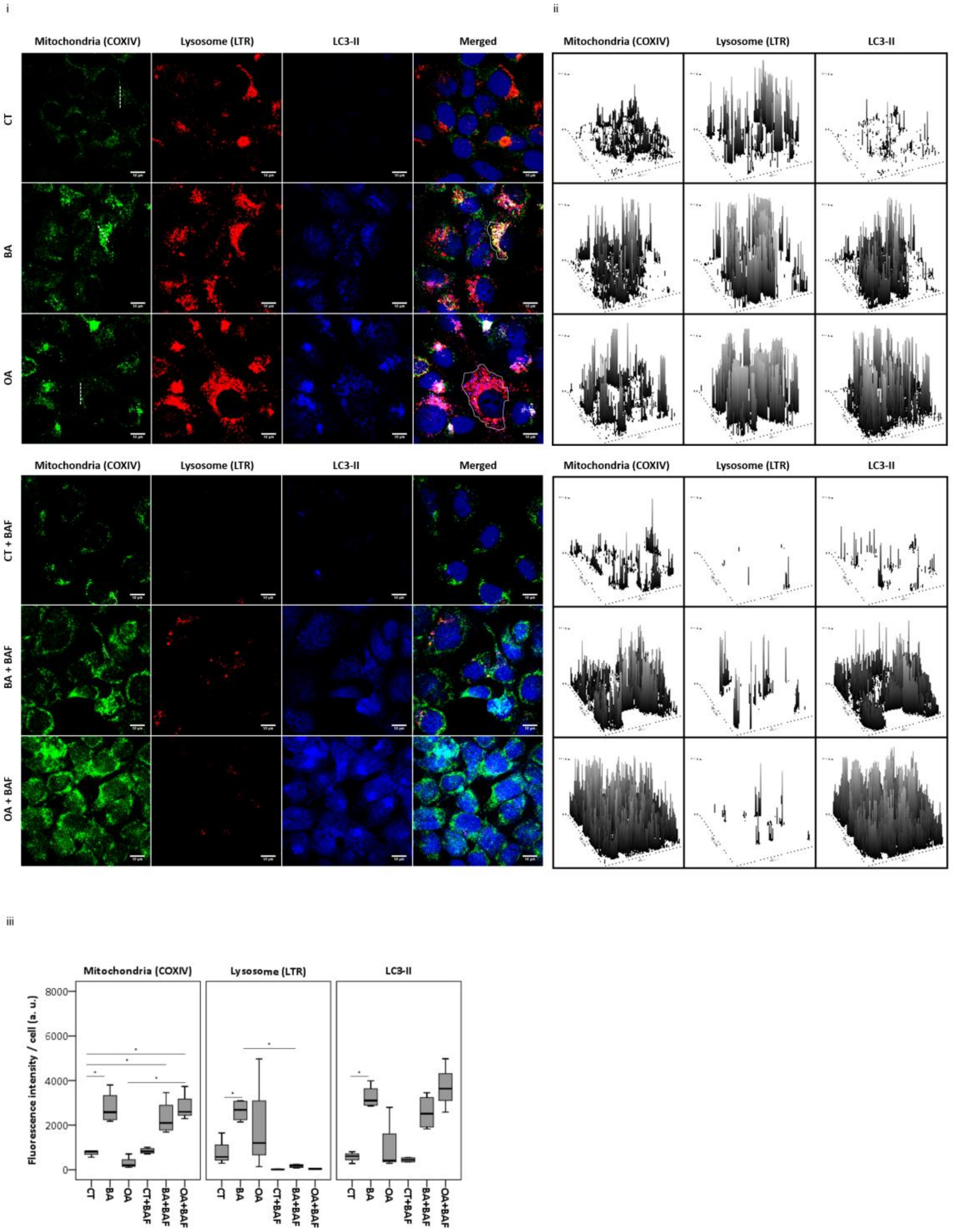

Supplementary Figure S1. Analysis of autophagic removal of mitochondria in HaCaT keratinocytes after treatment of triterpenoids. Micrographs of treated-cells at T1 with DMSO (control) and triterpenoids (20 µM) in presence or absence of BAF (2 ηM) treatment. Following staining of lysosomes with LTR (red), cells were immunostained for endogenous LC3-II (blue) and for the mitochondrial marker COXIV

(green). At right panel, we represented their respective surface plots (ii) and box-plots of multiple images (iii). All results obtained from at least three independent experiments and expressed as mean values ± standard error. Multiple statistical comparisons were calculated by ANOVA test, and the P value for each pairwise group was determined by Dunnett's T3 (high variance between groups) or Bonferroni (low variance between groups) post-hoc test. Significance difference ($p < 0.05$) was depicted by asterisk.

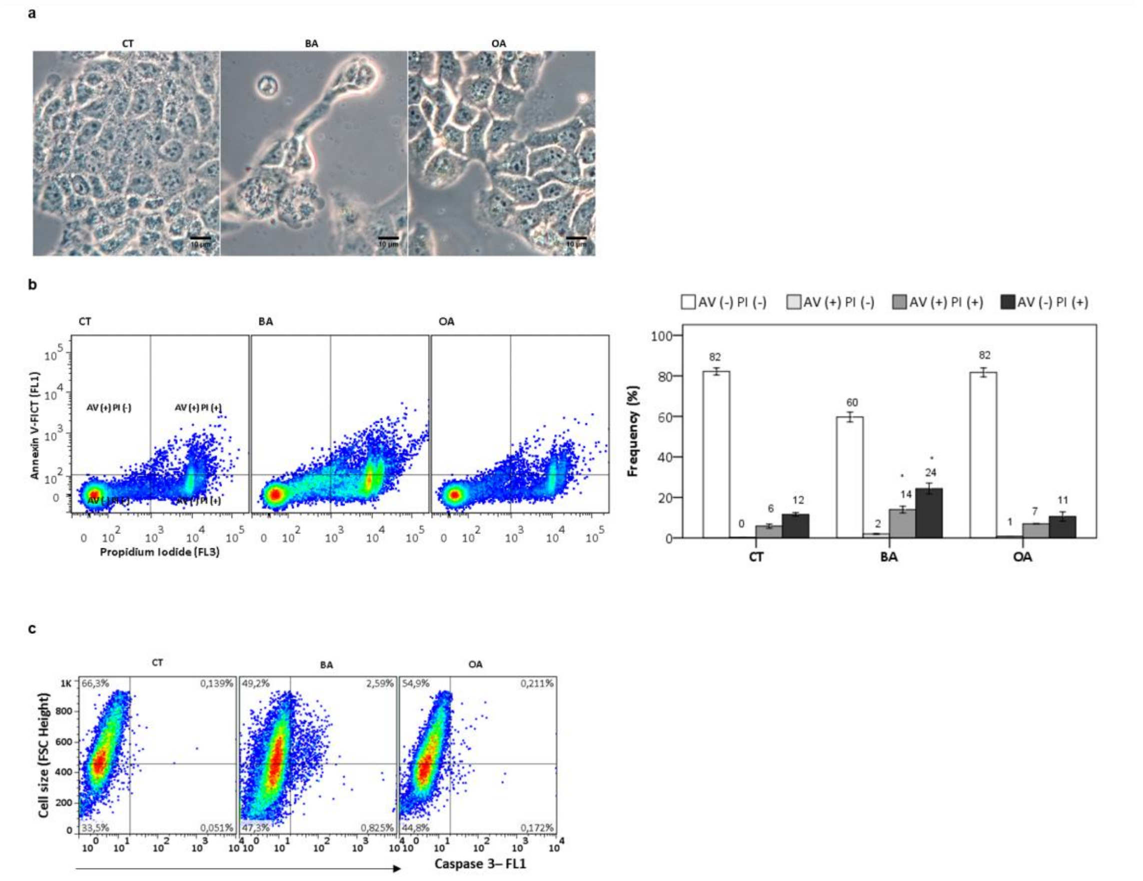

Supplementary Figure S2. Analysis of apoptosis in HaCaT keratinocytes after treatment of triterpenoids. (a) Micrographs of treated cells with DMSO (control) and triterpenoids (20 µM) at T1. (b) At T1 with DMSO (control) and triterpenoids (20 µM), following FACS treated cells were gated according to the parameters Annexin V (FL1) and Propidium Iodide (FL3). Representative pseudo-color scatter-plot showed the subpopulations [AV^-/PI^-; AV^+/PI^-; AV^+/PI^+; AV^-/PI^+] (left panel). The mean frequency of these subpopulations were represented by bars (right panel). (c) At T1 with DMSO (control) and triterpenoids (20 µM), following FACS the correlated distribution of treated cells according to the two parameters cell size and active Caspase-3 was represented by pseudo-color scatter-plots. In pseudo-color depiction, red represented higher frequency of cell population with indicative phenotype. All results obtained from at least three independent experiments and expressed as mean values ± standard error. Multiple statistical comparisons were calculated by ANOVA test, and the P value for

each pairwise group was determined by Dunnett's T3 (high variance between groups) or Bonferroni (low variance between groups) post-hoc test. Significance difference ($p < 0.05$) was depicted by asterisk.
